# Proteomic Basis of the Antibody Response to Monkeypox Virus Infection Examined in Cynomolgus Macaques and a Comparison to Human Smallpox Vaccination

**DOI:** 10.1371/journal.pone.0015547

**Published:** 2010-12-30

**Authors:** Sarah Keasey, Christine Pugh, Alexander Tikhonov, Gengxin Chen, Barry Schweitzer, Aysegul Nalca, Robert G. Ulrich

**Affiliations:** 1 United States Army Medical Research Institute of Infectious Diseases, Frederick, Maryland, United States of America; 2 Life Technologies, Carlsbad, California, United States of America; George Mason University, United States of America

## Abstract

Monkeypox is a zoonotic viral disease that occurs primarily in Central and West Africa. A recent outbreak in the United States heightened public health concerns for susceptible human populations. Vaccinating with vaccinia virus to prevent smallpox is also effective for monkeypox due to a high degree of sequence conservation. Yet, the identity of antigens within the monkeypox virus proteome contributing to immune responses has not been described in detail. We compared antibody responses to monkeypox virus infection and human smallpox vaccination by using a protein microarray covering 92–95% (166–192 proteins) of representative proteomes from monkeypox viral clades of Central and West Africa, including 92% coverage (250 proteins) of the vaccinia virus proteome as a reference orthopox vaccine. All viral gene clones were verified by sequencing and purified recombinant proteins were used to construct the microarray. Serum IgG of cynomolgus macaques that recovered from monkeypox recognized at least 23 separate proteins within the orthopox proteome, while only 14 of these proteins were recognized by IgG from vaccinated humans. There were 12 of 14 antigens detected by sera of human vaccinees that were also recognized by IgG from convalescent macaques. The greatest level of IgG binding for macaques occurred with the structural proteins F13L and A33R, and the membrane scaffold protein D13L. Significant IgM responses directed towards A44R, F13L and A33R of monkeypox virus were detected before onset of clinical symptoms in macaques. Thus, antibodies from vaccination recognized a small number of proteins shared with pathogenic virus strains, while recovery from infection also involved humoral responses to antigens uniquely recognized within the monkeypox virus proteome.

## Introduction

Human monkeypox is a zoonotic disease endemic in Central and West Africa [1]. The causative agent, monkeypox virus, belongs to the family Poxviridae, genus Orthopoxvirus. Of the seven known orthopox species, variola virus causes the most severe disease (smallpox) and various forms of the attenuated vaccinia virus are used for vaccination. Skin lesions and other early clinical manifestations of monkeypox in humans resemble those of smallpox [2]. In contrast to the human-specific host range of variola virus, rodents are thought to be a principal natural reservoir for the monkeypox virus and primates the incidental hosts of viral circulation [3]. Documented human-to-human spread of monkeypox [4] indicates the potential for natural selection of more virulent strains. Compared to smallpox, monkeypox is less contagious and is therefore geographically constrained. However, an outbreak of monkeypox occurred in the United States in 2003 resulting from the transmission of a West African strain of virus by rodents shipped from Ghana for the pet trade [5]. West African strains cause death in less than 1% of cases in Africa but there were no deaths occurring from the US outbreak and spread of human infection was rapidly contained. In contrast to West African strains, monkeypox viruses circulating in Central Africa are more virulent [6], [7], with case-fatality rates of approximately 10% among non-vaccinated individuals [8].

Despite the variability in host tropism and virulence, orthopox viruses exhibit a high degree of similarity in morphology, life cycle, and structure of the assembled virus. The approximately 200 kb of genomic DNA (double-stranded) encodes up to 280 genes, and replication of the morphologically distinct [9] intracellular mature virus (IMV) and extracellular enveloped virus (EEV) occurs within the host cell cytoplasm. The IMV has a physically-robust structure that facilitates transmission from host to host, while the more fragile EEV is encased by an envelope designed to limit host immune clearance and is thus adapted for intercellular spread of virus. The broad protection provided by vaccination indicates that orthopox viruses are antigenically related, and that exposure to one virus may protect from infection by another member of the family. The classical example of such protection is vaccination against variola (smallpox) by cowpox or vaccinia infection. Similarly, vaccination with vaccinia virus provided protection against monkeypox in a macaque model of disease [10], [11]. However, childhood smallpox immunization does not necessarily provide life-time protection from infection, as some vaccinated individuals may develop mild to moderate symptoms [12].

The worldwide human population is becoming increasingly susceptible to smallpox due to the end of routine vaccination in the 1970's, elevating concern for the increased incidence of monkeypox in Africa [13], potential emergence of new virulent strains, and the threat from bioterrorism. Because of these public health concerns, there is a need for better diagnostics as well as new safe and efficacious vaccines. Developing technological tools that bring a new perspective to our understanding of host responses to infectious diseases hasten the discovery of new vaccines or diagnostics. We, and others, have previously used whole proteome microarrays to measure antibody responses to individual proteins within the context of entire pathogen proteomes [14]–[16]. Here we describe a microarray containing nearly complete protein collections of both monkeypox and vaccinia viral proteomes, created with sequence-verified clones, purified protein components and high quality control. This orthopoxvirus protein microarray was used to examine potential relationships between antibody responses to monkeypox virus infection in cynomolgus macaques and smallpox vaccination in humans.

## Methods

### Required Ethics Statement

Peripheral arterial blood was collected from healthy human volunteers at the U.S. Army Medical Research Institute of Infectious Diseases (USAMRIID) for the preparation of serum, following written informed consent and in accordance with the protocol approved by the USAMRIID Institutional Review Board. Healthy, adult cynomolgus macaques (*Macaca fascicularis*) of both sexes were obtained from the USAMRIID animal colony. All animals exposed to monkeypox virus were handled in a BSL-3 containment laboratory at USAMRIID. Research was conducted in compliance with the Animal Welfare Act and other federal statutes and regulations relating to animals and experiments involving animals, and adhered principles stated in the Guide for the Care and Use of Laboratory Animals, National Research Council, 1996. The facility where this research was conducted (USAMRIID) is fully accredited by the Association for the Assessment and Accreditation of Laboratory Animal Care International. Research was conducted under a protocol approved by the Institutional Animal Care and Use Committee (IACUC) at USAMRIID. All animals were examined and evaluated twice per day by trained study personnel. Early endpoint criteria, as specified by the score parameters within the “Post-exposure observations” section of these methods, were used to determine when animals should be humanely euthanized to ameliorate any suffering.

### Disease and control sera

Cynomolgus macaques were challenged with controlled amounts of aerosolized monkeypox virus Zaire-1979_005, and clinical symptoms were monitored (Nalca et al. manuscript in preparation). The challenge resulted in lethal infections for animals that received the highest virus dose and minimal symptoms in those receiving the lowest dose. Four macaques challenged with an intermediate level of Zaire-1979_005 (5–100×10^4^ pfu) developed symptomatic monkeypox but fully recovered. We examined this intermediate challenge group in more detail. Serum samples were collected at days -1, 6, and 28 relative to the challenge date. Sera collected after challenges were gamma-irradiated (6 Mrad) to destroy any infectious virus. Previous results indicated minimal damage to serum immunoglobulins from the sterilizing dose of radiation (data not shown). Sera were also obtained from four human volunteers previously vaccinated with a smallpox vaccine (Dryvax,Wyeth) derived from the New York City Board of Health strain of vaccinia virus and from three naïve (non-vaccinated) control individuals. The serum samples from vaccinated individuals were collected one month after the last boost.

### Proteome microarrays

Proteins encoded by open-reading frames (ORFs) within the genomes of monkeypox and vaccinia viruses were produced as described previously [16]. Briefly, ORFs from monkeypox Zaire-1979_005 (DQ011155; 202 genes), WRAIR7-61 (AY603973; 178 genes), and vaccinia Copenhagen (M35027; 273 genes) were PCR-amplified, cloned and expressed in Sf9 insect cells using Gateway baculovirus expression (Invitrogen). All ORF clones were fully sequenced. Recombinant proteins carried GST-tags and were affinity purified using glutathione agarose. Purified orthopox proteins along with a series of positive and negative control proteins [16] were printed on thin-film nitrocellulose PATH slides (Gentel Biosciences). Protein spot densities of representative slides were measured by using an anti-GST antibody and compared to a dilution series of known quantities of protein also printed on each slide. Intra-slide and intra-lot variability in spot intensity and morphology, the number of missing spots and the presence of control spots were also measured and compared to a defined set of lot release standards before use in any reported studies. Based on preliminary assay optimization, microarrays were probed with serum diluted 1/150 (IgM detection in monkey sera and IgG in human sera) or 1/500 (IgG in monkey sera). Buffers and assay conditions were as previously described [15], [16]. Antibody binding was detected by incubation with Alexa-647 labeled goat anti-human IgG (H+L) and IgM (μ chain) at 1/2000 and 1/1000 dilutions, respectively.

### Data analysis

Digital images of antibodies interacting with the microarrayed proteins were collected using a GenePix Pro 6.0 (Molecular Devices) confocal laser scanner, and data were analyzed using ProtoArray Prospector 5.1 (Invitrogen) software (Table S1). The arrays were scanned (power = 100) using the highest PMT (photomultiplier tube) gain setting that did not produce signal saturation. An M-statistics algorithm (IRBP Toolbox v5.1, Invitrogen) was used to calculate significance of the results. Control and disease/vaccinated groups were created with the IRBP toolbox, and then compared by implementing a minimal signal of 500 relative fluorescence units (RFU) with a minimal gap of 200 RFU between signals to determine significant increases in fluorescent intensity among groups.

### Virus neutralization assay

Vero E6 cells (American Type Culture Collection, Manassas, VA) were suspended in EMEM/NEAA supplemented with 2% Fetal Bovine Serum (FBS), plated at a concentration of 5×10^5^ per well in a 12-well plate, and incubated for 12 h (37°C). One hundred plaque-forming units of Zaire-1979_005 (ZAIRE), obtained from cell lysate and supernatant from infected African green monkey kidney cells (American Type Culture Collection, Manassas, VA), were added (100 µl EMEM) to macaque sera serially diluted into 100 µl of EMEM and incubated (37°C, 1 h) with gentle rocking every 15 min. Medium was removed from each well and serum dilutions with virus were added (100 µl) to duplicate wells. Each culture plate also contained cell (media only) and virus (virus and media) control wells. The plates were mixed with gentle rocking every 15 min of incubation (37°C, 1 h) and 3 ml of plaquing media (1∶1 ratio of 2xEMEM/NEAA with 4% FBS and methyl cellulose) were added to each well for an additional 48 h. The plaquing media was removed and Crystal Violet with 20% MeOH was added to each well to stain for plaques. Plaques were counted and the 80% plaque reduction neutralization titer (PRNT80) was determined.

## Results

### Orthopox proteome microarrays

Gene insertions, truncations, duplications and substitutions of amino acid residues are common within genomes of monkeypox virus. For example, within the 194 genes of ZAIRE-96, 514 DNA sequence polymorphisms cause a change in amino acid composition when compared to all other complete monkeypox virus strains (average 2.6 amino acids/protein). Because it was not possible to include every protein variation expressed by all monkeypox virus strains, the microarray was designed to contain proteins from two monkeypox strains, ZAIRE and WRAIR7-61 (WRAIR), representing Central and West African clades, respectively (Table 1). Also included in the microarray were proteins from a commonly used vaccine strain, vaccinia Copenhagen (VACCOP). In general the orthopoxvirus strains represented on the array exhibit a high degree of protein sequence similarity, with ZAIRE and WRAIR sharing 62 identical proteins. However, only four proteins are identical among all three strains. For consistency, we use VACCOP nomenclature for orthologous proteins encoded by monkeypox virus genomes, and vaccinia Western Reserve for ORFs missing from VACCOP. We attempted to express all non-identical proteins from each strain because it was not possible to predict how minor differences in amino acid sequence translated into significant alterations of antibody recognition. All ORF clones isolated for protein expression were sequence-verified throughout the entire insert length and shuttled into bacmids. Protein production in eukaryotic cells, via conversion of bacmid clones into baculovirus for insect cell expression, increased the likelihood that all products were correctly folded and functional. Successfully cloned, expressed and size-verified proteins were contact printed on nitrocellulose-coated glass slides. Statistical samplings of each printed lot of microarrays were evaluated for quality and consistency before use in experiments. Combined coverage of the vaccinia and monkeypox proteomes on the microarrays was 92–95%.

**Table 1:** Orthopoxvirus microarray proteome coverage.*

Strain	GenBank Accession #	Proteins				
		Total encoded within genome	Identical to VACCOP	Identical to ZAIRE	Identical to WRAIR	Printed (% coverage)
VACCOP	M35027	273	-	4	4	250 (92)
ZAIRE	DQ011155	202	4	-	62	192 (95)
WRAIR	AY603973	178	4	62	-	166 (93)

*Protein numbers and identity based on the poxvirus database annotations ( www.poxvirus.org **)**.

### Detection of antibody responses to pre-symptomatic monkeypox

**Figure 1:**
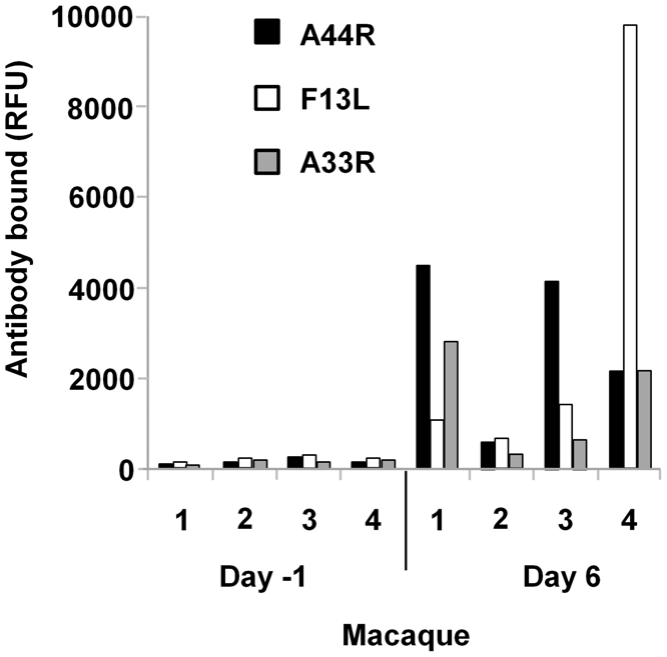
Acute phase IgM binding to ZAIRE proteins. Interactions of arrayed orthopox virus proteins with antibody from serum collected from four macaques (1–4) before (−1) and 6 days after infection with monkeypox virus. Data are presented as mean fluorescent intensity signal (mean RFUs of 2–4 protein spots). SD<15%.

Monkeypox is a potentially fatal disease for cynomolgus macaques. However, challenge with sublethal levels of ZAIRE virus resulted in a fully-symptomatic disease that was successfully resolved solely by the immune response of the host without medical intervention. We examined sera collected before viral challenge and at several time points leading up to full recovery from disease, reasoning that this surviving subject group should harbor the most robust antibody response against monkeypox virus. Mild anorexia, depression and fever were observed in macaques no earlier than 6 days after initial infection. Skin rashes were initially observed beginning on day 8 (amount was independent of viral challenge dose) and progressed from macules to papules, then vesicles and pustules to scabs over 10 days. Lymphadenopathy, which differentiates monkeypox from smallpox, was first observed in macaques no earlier than 6 days after initial infection. We first examined the antibody response during the earliest phase of infection just prior to onset of disease symptoms by probing the microarrays with sera collected before (day -1) and 6 days after challenge. Using group data analysis (M-statistics), we detected increased binding to select arrayed proteins by IgM from the day 6 sera compared to the day -1 sera across all animals in the group, while IgG interactions with the orthopox proteins were negligible. We confirmed significant (p≤0.01) IgM binding to 71 individually arrayed proteins (58 orthologs) in the day 6 sera compared to the pre-challenge, with 19 proteins exhibiting more than a fivefold average signal increase (Table 2). Further, three ZAIRE proteins (Figure 1) exhibited more than a tenfold increase in IgM binding for day 6 compared to pre-challenge sera: A44R, an 8.5 Kd protein of unknown function and the VACCOP envelope proteins F13L and A33R, both expressed by EEV. Approximately half of the proteins recognized by IgM were structural elements (envelope or core proteins).

**Table 2:** Orthopoxvirus proteome recognized by IgM during acute infection.

Vaccinia ortholog	Predicted function	Fold increase in antibody a		
		VACCOP	VirusZAIRE	WRAIR
F13L	EEV phospholipase, envelope protein	14	16	16^d^
A44R	unknown	5	19	11
A33R	EEV envelope protein	7	11	11^d^
A46R	Toll-IL1-receptor-like protein	5	10	10
C23L	chemokine binding protein	7	9	8
A48R	thymidylate kinase	5	9	7
B5R	EEV envelope protein	7	6	6 d
J1R	virion protein required for morphogenesis	9	6	3
E11L	core protein	7	5	5^d^
A4L	core protein	5	5	6
B15R	unknown	7	4	5
E3L	dsRNA binding protein	7	6	2
WR_146	A-type inclusion protein, fragment	NA^b^	5	NP^c^
H3L	IMV envelope protein	4	5	5
K7R	unknown	NP	3	5
B11R	unknown	5	2	5
F12L	IEV surface protein	2	5	NP
C16L	unknown	3	2	5
A26L	IMV envelope protein	1	3	5

a Proteins exhibiting more than a fivefold average signal increase. Average signals from sera collected at day 6 normalized to average signal at day -1.

b NA, not present in genome,

c NP, not present on array.

d 100% identity between WRAIR and ZAIRE proteins.

### Convalescent IgG responses

We next examined the antibody response following full recovery from infection by probing the orthopox microarrays with sera collected before (day -1) and 28 days after challenge, examining binding of IgG to the orthopoxvirus proteome. There was no IgM or IgA binding detected with the convalescent sera (data not shown), indicating that only IgG responses were measurable. Significant interactions between IgG and 23 independent orthopoxvirus proteins were detected (Table 3) in convalescent sera compared to normal sera (M-statistics; minimal signal and minimal difference of 500 RFUs). The greatest level of IgG binding occurred with the EEV structural proteins F13L and A33R, and the IV membrane scaffold protein D13L. There were two non-structural proteins with relatively high immunogenicity: WR_148, a protein of unknown function, and the transcriptional factor H5R. While the IgM response to F13L and A33R that was detected in acute-phase sera progressed to a convalescent-IgG response, no significant IgG binding to A44R was detected in sera from recovered macaques. The biological significance of the diminished IgG response to A44R is unknown.

**Table 3:** Orthopoxvirus proteome recognized by IgG in convalescent serum.

Vaccinia ortholog	Predicted function	Fold increase in antibody a		
		VACCOP	VirusZAIRE	WRAIR
F13L	EEV envelope protein	387	448	448^b^
D13L	IV membrane scaffold protein	294	207	72
A33R	EEV envelope protein	56	211	211^b^
WR_148	A-type inclusion protein, fragment	NA^c^	119	NP^d^
D8L	IMV envelope protein	116	161	47
A4L	core protein	113	99	88
A27L	IMV envelope protein	36	111	149
H3L	IMV envelope protein	130	66	93
I1L	core protein	81	91	91^b^
H5R	transcription factor	80	101	70
A10L	core protein	124	86	30
E3L	dsRNA binding protein	9	101	37
A26L	IMV envelope protein	6	86	2
C13L/C14L^e^	unknown	2/4	62	18
B5R	EEV envelope protein	46	33	33^b^
B13R/B14R^e^	serine proteinase inhibitor	3/4	10	49
B19R	IFN-alpha/beta receptor	8	37	15
A46R	Toll-IL1-receptor [TIR]-like protein	4	28	35
B2R/B3R^e^	Schlafen	4/2	21	10
C23L	chemokine binding protein	17	11	10
L4R	core protein	14	10	10^b^
B1R	Ser/Thr kinase	20	1	1
F17R	core protein	6	5	5^b^

a Average signals from sera collected at day 28 normalized to average signal at day -1.

b 100% identity between WRAIR and ZAIRE proteins.

c NA, not present in genome.

d NP, not present on array.

e VACCOP proteins orthologous to fragments of a monkeypox protein.

### Neutralizing antibody and the monkeypox virus proteome

**Figure 2:**
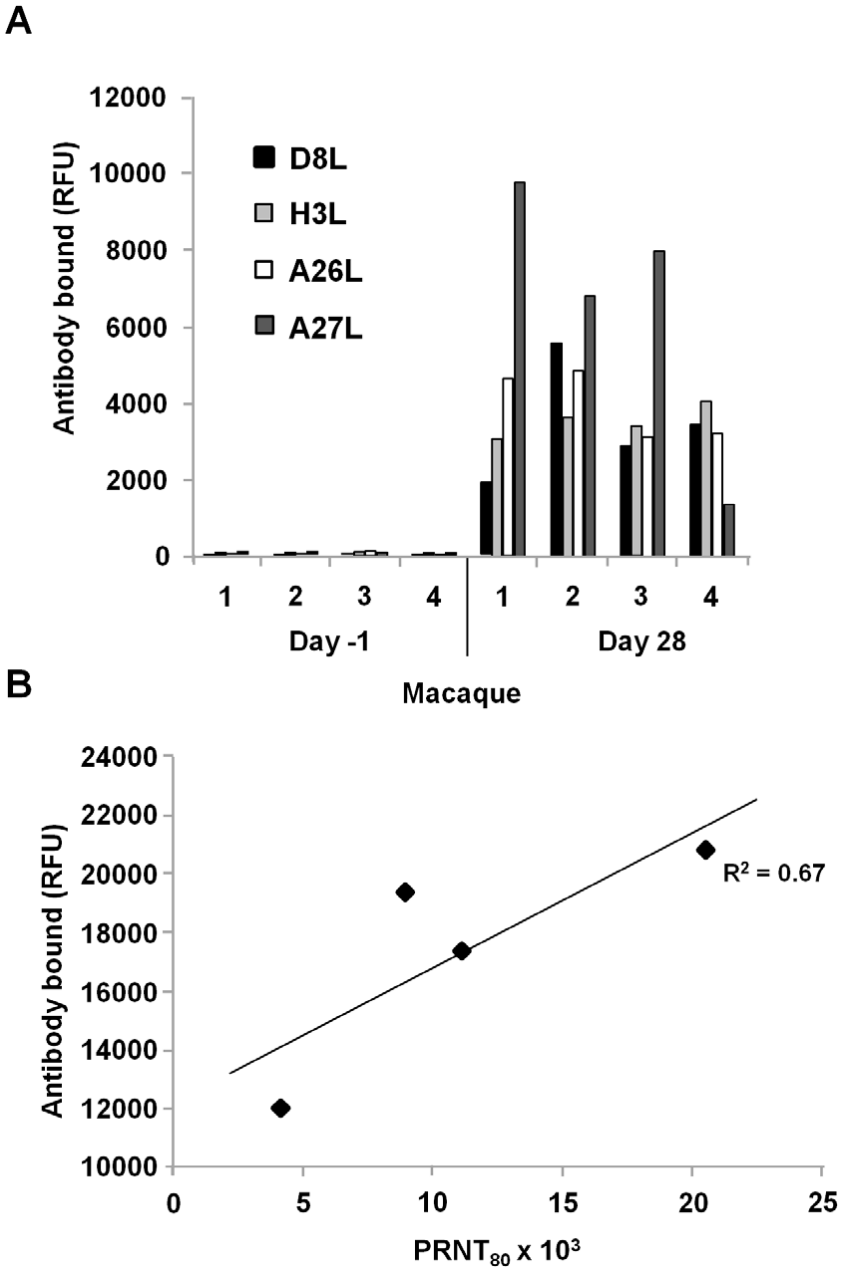
IgG responses to ZAIRE IMV envelope proteins contribute to virus neutralization. (A) Antibody binding at days -1 and 28 relative to viral challenge in four macaques (1–4). Mean RFUs of 2–4 protein spots (SD<15%). (B) Correlation between IgG binding to IMV and serum neutralizing activity. Total antibody binding level to the IMV surface was estimated as the sum of fluorescent intensities for A26L, A27L, D8L, and H3L and analyzed for association with PRNT80 using linear regression.

The results measured up to this point presented a static profile of proteins that stimulated IgG and IgM responses. To better understand the physiological relevance of these protein antigens we examined the neutralization of virus infection in cell culture by the immune sera, using an assay of plaque-reducing neutralizing titers (PRNT), focusing on convalescent antibodies. The preparation of ZAIRE virus for the PRNT assay favored preservation of predominantly the IMV form due to the fragility of EEV membranes [9]. Since neutralizing antibodies were expected to bind mostly IMV surface proteins, we examined IgG in convalescent sera interacting with the IMV proteins D8L, H3L, A26L, and A27L (Figure 2A). The total antibody binding of these IMV surface proteins (Figure 2B) demonstrated a linear relationship with PRNT80 (R^2^ = 0.67), while interactions of the individual proteins demonstrated a weaker correlation (R^2^ = 0.05–0.53). Therefore, the data suggested that antibodies to the four IMV proteins contribute to virus neutralization. Future studies in animal models may allow broader conclusions regarding the physiological relevance of these specific antibody responses to blocking the spread of infective virus.

### Human IgG recognition of the orthopoxvirus proteome

From data obtained with a microarray comprised of the vaccinia virus proteome, we previously reported [16] that human vaccination resulted in the induction of IgG that was specific for eight vaccinia proteins: H5R, C3L, I3L, A27L, D13L, I1L, H3L, and A33R. Because smallpox vaccination was previously noted to provide immunity to infections caused by monkeypox virus, we examined cross-reactivity of the humoral response developed in humans vaccinated with the Dryvax preparation of vaccinia virus. Four individuals received two or more vaccinations, with the last boost occurring one month prior to serum collection. Sera from unvaccinated individuals (n = 3) were used for controls. Collectively, IgG from these immune sera recognized 14 proteins of monkeypox virus on the microarray (p≤0.03;M-statistics minimal signal of 500, difference of 200) as presented in Table 4. Notably, all proteins recognized by IgG from vaccinated humans (Table 4) were also recognized by macaque serum IgG (Table 3), except for two proteins with the lowest significant signal: D11L and I4L. While serum from some vaccinated individuals exhibited significant PRNT data, overall these results were too low for interpretation, perhaps due to the reduced ability of anti-vaccinia antibodies to bind to the heterologous monkeypox virions.

**Table 4:** Monkeypox virus proteome recognized by IgG from vaccinated humans.

Vaccinia ortholog	Predicted function	Fold increase in antibody a	
		Virus	
		ZAIRE	WRAIR
F13L	EEV envelope protein	59	59^b^
H3L	IMV envelope protein	23	19
I1L	DNA binding core protein	55	55^b^
D13L	IV membrane scaffold protein	28	6
A10L	core protein	24	17
A33R	EEV envelope protein	17	17^b^
A26L	IMV envelope protein	20	1
WR_148	A-type inclusion protein, fragment	12	NP^d^
A4L	core protein	9	11
B5R	EEV envelope protein	4	4^b^
D8L	IMV envelope protein	4	3
B2R/B3R^e^	Schlafen	5	1
D11L	nucleoside triphosphate phosphohydrolase	2	2
I4L	ribonucleoside reductase	2	2

a Average signals in sera of vaccinated normalized to signals in normal sera.

b 100% identity between WRAIR and ZAIRE proteins.

c NA, not present in genome.

d NP, not present on array.

e VACCOP proteins orthologous to fragments of a monkeypox protein.

### Antibody recognition by protein abundance

**Figure 3:**
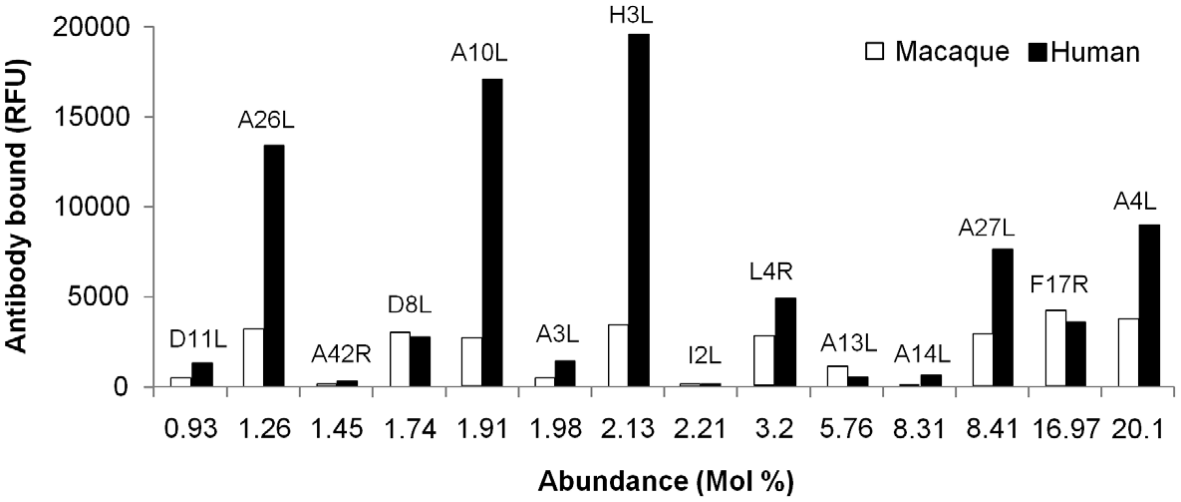
Relationship between antibody responses and abundance of IMV proteins. Relative levels of antibody binding were plotted against the most abundant proteins of vaccinia IMV [refs 17–20], using convalescent macaque and vaccinated human sera.

All but three (B1R, B2R, and WR_148) of the 25 IgG binding proteins identified in our study were previously reported in IMV or EEV (Table 5), as measured by mass spectrometry [17]–[20]. Nine of the 15 most abundant proteins in the vaccinia IMV, based on mole % [17], [18], demonstrated significant antibody binding (Table 5). While no correlation (R^2^<0) between level of binding (RFUs) and protein abundance was found (Figure 3), there was a trend between higher abundance and antibody recognition.

**Table 5:** Antibody (IgG) responses of convalescent macaque and vaccinated human compared to relative abundance of viral protein.

Orthopoxvirus^1^ protein	Predicted Function	Known ZAIRE^2^	abundance VACCOP
**A4L**	core protein	**+**	**+**
**A10L**	core protein	**+**	**+**
**A26L**	IMV envelope protein	**+**	**+**
**A27L**	IMV envelope protein	**+**	
A33R	EEV envelope protein	**+**	**+**
A46R	Toll-IL1-receptor-like protein	**+**	
B1R	ser/thr kinase	**+**	
B2R/B3R^3^	Schlafen	**+**	**+**
B5R	EEV envelope protein	**+**	**+**
B13R/B14R^3^	serine proteinase inhibitor	**−**	
B19R	IFN-alpha/beta receptor	**−**	
C13L/C14L^3^	unknown	**−**	
C23L	chemokine binding protein	**−**	
**D8L**	IMV envelope protein	**+**	**+**
**D11L**	nucleoside triphosphate phosphohydrolase		**+**
D13L	IV membrane scaffold protein	**+**	**+**
E3L	dsRNSA binding protein	**+**	
F13L	EEV phospholipase, envelope protein	**+**	**+**
**F17R**	core protein	**+**	
**H3L**	IMV envelope protein	**+**	**+**
H5R	transcription factor	**+**	
I1L	core protein	**+**	**+**
I4L	ribonucleoside reductase		**−**
**L4R**	core protein	**+**	
WR_148	A-type inclusion protein, fragment	**+**	**+**

1 Bolded orthopox proteins: identified by mass spectometry as one of 15 most abundant proteins, based on mole percent [17].

2 Antibody-binding protein, no abundance data available (−). Antibody-binding protein previously measured [17]–[20] by mass spectrometry (+).

3 Vaccinia virus protein similar to protein of monkeypox virus.

## Discussion

Global vaccination with live vaccinia virus resulted in the successful eradication of smallpox, with Somalia reporting the last naturally occurring case in 1977. Under pressure from vaccination, the specificity of variola virus for humans and the absence of animal or environmental reservoirs were also significant factors that helped eradicate smallpox. The zoonotic origin of monkeypox raises concern that the virus may evolve to become more dangerous for human populations despite current levels of relatively low transmissibility and virulence. Vaccination against smallpox reduces lesions and the severity of monkeypox [1], [11], indicating a significant level of cross-reactive immunity. Our results serve to define the proteomic basis of this common antibody response to infection by monkeypox virus and smallpox vaccination. By examining antibodies from ZAIRE-infected macaques we observed extensive cross-reactivity between the proteomes of ZAIRE and WRAIR strains, but could not establish any basis for clade-specific immunity in terms of antibody responses. This is noteworthy because the distinct virulence and transmissibility exhibited by the Central and West African monkeypox viruses represented on the microarrays used in our study may be independent of host antibody responses. Previous genomic comparisons between these two dominant clades [6], [7], [21] identified several protein candidates that may increase virulence or alter host immunity thereby leading to differences in clinical outcome. However, due to the apparent similarity in humoral responses to monkeypox viruses, our results suggest that further attenuation of WRAIR or a similar West African strain of virus may provide broader immunity against severe monkeypox than currently used vaccinia-based vaccines. While our data collected with proteome microarrays presents only one facet of poxvirus immunity, these antibody-mediated responses are essential because prior studies in macaques demonstrated that depletion of B cells, but not CD4+ or CD8+ T cells, reversed the protection provided by vaccinia-virus immunity, resulting in lethal infection by monkeypox virus [11].

Detection of IgM specific to infection has important diagnostic implications because it can be used to distinguish acute monkeypox from the antigen-specific IgG resulting from previous smallpox vaccinations. The estimated mean human incubation period for monkeypox virus infection is 12 days [5] and the optimal timing of specimen collection for determination of IgM levels using current assays is 7–56 days after appearance of a rash [22]. Detection of IgM responses earlier than 4 days after rash onset was problematic in an earlier study that examined monkeypox viral infection in humans using whole virus in an ELISA [23]. Our data indicate the possibility of orthopox-specific IgM detection even before rash onset. In addition, IgM detection using individual monkeypox proteins in a microarray format may substantially increase assay sensitivity compared to a whole virus ELISA. We detected specific IgM responses to monkeypox virus by 6 days after infection, though earlier time points of sample collection were not included in the study. Three monkeypox proteins, orthologs of the vaccinia virus proteins F13L, A33R, and A44R, exhibited substantial (>10×) increases in IgM binding in the day 6 sera compared to pre-challenge levels. While F13L and A33R are well-known vaccinia EEV envelope antigens, antibody recognition of A44R is a new finding. The protein A44R is a 38 amino acid residue fragment of VACCOP (Table 6), whereas the longer ZAIRE and WRAIR orthologs are closer in length to the A44R protein of vaccinia Western Reserve, WR_169 (VACCWR). Though the function of this protein is unknown, A44R contains strings of lysine and aspartic acid residues that suggest a stable hairpin fold created by salt bonding. About half of the IgM-binding proteins detected six days after monkeypox challenge were also recognized by IgG from convalescent sera. Interestingly, no IgG response was detected against A44R. Clearly, F13L, A33R, and A44R are potential candidates for diagnostic tests of acute monkeypox infection (Table 2).

**Table 6:** Protein A44R of monkeypox and vaccinia viruses.

Virus	Amino acid sequence
VACWR	MLLEMDKIKITVDSKIGNVVTISYNLEKITIDVTPKKKKEKDVLLAQSVAVEEAKDVKVEEKNIIDIEDDDDMDVESA
VACCOP	MDKIKITVDSKIGNVVTISYNLEKITIDVTPKKKRMYY
ZAIRE	MDKIKITIDSKIGNVVTISYNLEKITIDVTPKKKKEKDVLLAQSVAVEEAKDVKVEEKNIIDIEDDDDMDIENT
WRAIR	MDKIKITIDSKIGNVVTISYNLEKITIDVAPKKKKEKDVLLAQSVAVEEAKDVKVEEKNIIDIEDDDDMDIENT

The majority of IgG-binding proteins observed in our study were previously reported to be present in high amounts within assembled virions [17]–[20], though we cannot conclude that abundance alone favors antibody responses. Previous studies reported protein targets for neutralizing antibodies on the IMV surface of vaccinia virus [24], [25]. In the results presented here, we also observed a correlation between the antibody response to the IMV proteins A26L, A27L, D8L, and H3L with monkeypox virus neutralization by sera from ZAIRE-infected macaques (Figure 2A). Further, humans vaccinated with Dryvax demonstrated IgG responses to the orthopoxvirus proteome that were similar to those obtained from the serum of macaques recovering from monkeypox. There were 12 of 14 antigens detected in human vaccinees that were also recognized by IgG from convalescent sera of macaques, whereas serum IgG of macaques that recovered from monkeypox recognized at least 23 separate proteins within the orthopox proteome. More viral proteins were recognized by macaque than human antibodies, perhaps because monkeypox produced a more intense immune response than human vaccination. Further, our data compared aerosol challenge in macaques with human skin vaccination. It is possible that antibodies from humans recovering from monkeypox could recognize an increased number of antigens compared to vaccination, though we have not had the opportunity to test this hypothesis. It should also be noted that group statistical methods were used to analyze our microarray results, and all significant antibody interactions that were reported occurred in a majority of the subjects examined. We did not consider the potential contribution of variability among individuals in antibody responses, though this is likely to influence the outcome of infection. Missing from vaccinia strains, the large immunogenic surface glycoprotein B21R of monkeypox viruses may be useful for distinguishing monkeypox from previous vaccinia vaccination [26]. However, we did not examine B21R in our study because the corresponding ZAIRE and WRAIR orthologs were not stably expressed by insect cells and were therefore missing from the microarrays. Further, the insect cells we used for protein production synthesize mostly simple N-glycans with terminal mannose residues [27], while antibody recognition of the poxvirus products may be influenced by host protein glycosylation. Regardless of the glycosylation pattern, the immune response to saccharides predominantly results in the production of IgM antibody with little memory component [28], and is thus more likely to impact analysis of serological responses only during the acute phase of infection.

There is substantial evidence that antibody responses resulting from vaccination play a central role in protection against poxvirus infections. However, the safety and efficacy of current vaccines are still significant concerns. High-throughput approaches based on microarrays of the vaccinia virus proteome were recently used to identify or confirm individual viral proteins associated with smallpox immunity [14], [16]. In addition, these studies revealed that antibody responses were directed towards a small subset of antigens contained within the viral proteome [16] and that the total number of viral proteins recognized by antibodies varied from person to person, perhaps because specificities of antibody and CD4+ T cell helper responses were found to be tightly linked and influenced by HLA class II polymorphism [29]. The technical advantage that protein microarrays provide to capturing interactions between pathogen and human hosts at the proteomic scale will prove increasingly more important to understanding the emergence of new infectious diseases and for devising methods for medical intervention.

## Supporting Information

Table S1 **Proteome Microarray Data (Relative Fluorescent Units).**
(PDF)
