# Supplementary material for: Proteomic Basis of the Antibody Response to Monkeypox Virus Infection Examined in Cynomolgus Macaques and a Comparison to Human Smallpox Vaccination
Source: PLoS One. 2010 Dec 30;5(12):e15547. doi: 10.1371/journal.pone.0015547 (PMC3012712; doi:10.1371/journal.pone.0015547)
Supplement: Table S1 — Proteome Microarray Data (Relative Fluorescent Units). (PDF) [file pone.0015547.s001.pdf]

Vaccinia Ortholog	Database ID		Control Non-Human Primates: Day -1 (IgG)																	
			1	2	3	4	5	6	7	8	9	10	11	12	13	14	15	16	17	18
A4L	MPXV_ZAI1979_120		374.5	441	551.5	444	303	822	98	76	315.5	368.5	1638.5	149.5	263	303.5	266	266	663.5	2544
	MPXV_ZAI1979_120		451.5	471.5	531	383.5	305.5	841	104	85.5	291	446	1648.5	142	279	318.5	304	237.5	695.5	2402
		average of replicates	413	456.25	541.25	413.75	304.25	831.5	101	80.75	303.25	407.25	1643.5	145.75	271	311	285	251.75	679.5	2473
		**average all**	**550.70833**																	
A4L	MPXV-WRAIR109		430	528	384.5	546	301.5	824	132	113.5	386.5	461	1513.5	191.5	284	338.5	395	321	636.5	1989
	MPXV-WRAIR109		461	483	461	615	331	792	148.5	107	486	547.5	1339	168	288	354	340.5	310.5	440.5	2085
		average of replicates	445.5	505.5	422.75	580.5	316.25	808	140.25	110.25	436.25	504.25	1426.25	179.75	286	346.25	367.75	315.75	538.5	2037
		**average all**	**542.59722**																	
A10L	MPXV_ZAI1979_126		228	1.5	304.5	502.5	384.5	303.5	401	314	546	502.5	262	467	294.5	304	369.5	333	268	274.5
	MPXV_ZAI1979_126		362	290	308	549.5	351.5	397.5	373	294.5	733	505	250.5	515	303.5	248	325.5	340	302	285
	MPXV_ZAI1979_126		199	181.5	204	365	276.5	260	421.5	302.5	212.5	299	180.5	546	169.5	126.5	156	257.5	800	286.5
	MPXV_ZAI1979_126		254.5	167	224.5	431.5	266	249	417	324.5	149.5	340.5	161.5	539	171.5	153.5	164	261	196	275
	MPXV_ZAI1979_126		11	94.5	100.5	106.5	139.5	138	155	145.5	84	195.5	87.5	178	78.5	117.5	50.5	128	44	137.5
	MPXV_ZAI1979_126		2667	87.5	88	135.5	149.5	126.5	192.5	139	84	169.5	106.5	180	82.5	103.5	107	146	23.5	135.5
		average of replicates	620.25	137	204.91667	348.41667	261.25	245.75	326.66667	253.33333	301.5	335.33333	174.75	404.16667	183.33333	175.5	195.41667	244.25	272.25	232.33333
		**average all**	**273.13426**																	
A26L	MPXV_ZAI1979_146		337.5	339	274	371.5	362.5	304	390.5	335.5	385	281.5	342.5	319	301.5	213.5	217.5	324.5	247	356.5
	MPXV_ZAI1979_146		388	358.5	274.5	319	397.5	288	397.5	294.5	398	211	310.5	316.5	304	237.5	229	319	399	345.5
		average of replicates	362.75	348.75	274.25	345.25	380	296	394	315	391.5	246.25	326.5	317.75	302.75	225.5	223.25	321.75	323	351
		**average all**	**319.18056**																	
A27L	MPXV_ZAI1979_147		1376	1109	318.5	931.5	489.5	268.5	305.5	286	229	658	395	194.5	380.5	677	468	393	1183	542.5
	MPXV_ZAI1979_147		1001.5	1207	312	1056	442	261	266	310	279	893.5	380.5	211	299	587.5	475.5	449.5	1263	583
		average of replicates	1188.75	1158	315.25	993.75	465.75	264.75	285.75	298	254	775.75	387.75	202.75	339.75	632.25	471.75	421.25	1223	562.75
		**average all**	**568.94444**																	
A27L	MPXV-WRAIR132		228	837	253.5	547	371	282	284	423.5	190	443.5	362.5	205	285	356.5	294.5	396	480	502.5
	MPXV-WRAIR132		375.5	792	302	561	348.5	278.5	273	420.5	274	433	353	244	262	339	275	388.5	302	541
		average of replicates	301.75	814.5	277.75	554	359.75	280.25	278.5	422	232	438.25	357.75	224.5	273.5	347.75	284.75	392.25	391	521.75
		**average all**	**375.11111**																	
D8L	MPXV_ZAI1979_110		372.5	153	130.5	100.5	279.5	199	185.5	201	237.5	147.5	147.5	201	155.5	193.5	191	185.5	137.5	192.5
	MPXV_ZAI1979_110		469	148.5	130.5	67.5	260	191.5	161.5	176.5	152	139	167.5	157.5	143	200	191.5	169.5	327.5	192.5
		average of replicates	420.75	150.75	130.5	84	269.75	195.25	173.5	188.75	194.75	143.25	157.5	179.25	149.25	196.75	191.25	177.5	232.5	192.5
		**average all**	**190.43056**																	
D8L	MPXV-WRAIR099		401	549	811.5	745	767	868	893	834.5	626	721	505	952	503.5	663.5	541	857	587.5	745
	MPXV-WRAIR099		464	512	845.5	572	762.5	841.5	857	796	611.5	685	615	823.5	558.5	578.5	594.5	799	510.5	785
		average of replicates	432.5	530.5	828.5	658.5	764.75	854.75	875	815.25	618.75	703	560	887.75	531	621	567.75	828	549	765
		**average all**	**688.38889**																	
D11L	MPXV_ZAI1979_113		1014	439.5	561	1777	591	505	764.5	438.5	598	763	956.5	588.5	834.5	1168	1382.5	1271.5	1952	761
	MPXV_ZAI1979_113		1311.5	399	615	1902.5	572	591	666.5	451.5	512	625	890.5	559.5	704.5	1307	1505	608.5	2207.5	636.5
		average of replicates	1162.75	419.25	588	1839.75	581.5	548	715.5	445	555	694	923.5	574	769.5	1237.5	1443.75	940	2079.75	698.75
		**average all**	**900.86111**																	
F17R	MPXV_ZAI1979_052		7084.5	4993	4748.5	7084.5	3809.5	2640.5	3277	1459.5	4250	7084.5	6112	1476.5	3707.5	7084.5	7084.5	3186	7084.5	5741.5
	MPXV_ZAI1979_052		6629.5	5214.5	4577	6629.5	4089.5	2819.5	3174.5	1598	4577	6629.5	5829	1617.5	3915.5	6629.5	6629.5	3317.5	6629.5	5921.5
		average of replicates	6857	5103.75	4662.75	6857	3949.5	2730	3225.75	1528.75	4413.5	6857	5970.5	1547	3811.5	6857	6857	3251.75	6857	5831.5
		**average all**	**4842.6806**																	
H3L	MPXV_ZAI1979_098		481.5	428.5	285	501.5	365	428.5	395	304	443.5	389.5	415.5	410	852.5	415.5	380.5	441	307	396
	MPXV_ZAI1979_098		227.5	340.5	367	483	440	464	400	333	475.5	352	451	454.5	859	383.5	362.5	478	331.5	419
		average of replicates	354.5	384.5	326	492.25	402.5	446.25	397.5	318.5	459.5	370.75	433.25	432.25	855.75	399.5	371.5	459.5	319.25	407.5
		**average all**	**423.93056**																	
H3L	MPXV-WRAIR087		685	528.5	625	956.5	655	723.5	824.5	802.5	873.5	557	754.5	735	804.5	736.5	669.5	736.5	802	626
	MPXV-WRAIR087		790.5	534.5	622.5	873.5	675	705.5	775.5	775.5	1008.5	650	685	764.5	820	730	653	745	762	637
		average of replicates	737.75	531.5	623.75	915	665	714.5	800	789	941	603.5	719.75	749.75	812.25	733.25	661.25	740.75	782	631.5
		**average all**	**730.63889**																	
L4R	MPXV_ZAI1979_088		1695	2728	2842	2536.5	2900	2673.5	3244	3186	3244	2633.5	2842	3447.5	2492	2603.5	2387.5	2819.5	2218	2652.5
	MPXV_ZAI1979_088		1533	2544	2857	2359.5	2857	2622	3186	3174.5	3121	2603.5	2728	3317.5	2544	2424.5	2133	2791.5	1879.5	2791.5
		average of replicates	1614	2636	2849.5	2448	2878.5	2647.75	3215	3180.25	3182.5	2618.5	2785	3382.5	2518	2514	2260.25	2805.5	2048.75	2722
		**average all**	**2683.6667**																	

Vaccinia Ortholog	Database ID		Non-Human Primates Day 6 (IgG)																	
			1	2	3	4	5	6	7	8	9	10	11	12	13	14	15	16	17	18
A4L	MPXV_ZAI1979_120		781	223	1118	649	270	444	137	124	145	286	357	285	267	231	370	553	450	499
	MPXV_ZAI1979_120		941	215	1182	664	186	283	142	135	141	294	372	292	267	198	384	528	453	481
		average of replicates	861	219	1150	656.5	228	363.5	139.5	129.5	143	290	364.5	288.5	267	214.5	377	540.5	451.5	490
		**average all**	**398.5278**																	
A4L	MPXV-WRAIR109		481	279	952	562	288	450	254	169	173	274	432	227	238	225	373	599	489	464
	MPXV-WRAIR109		467	283	912	541	282	423	214	178	220	342	441	254	249	210	345	537	468	474
		average of replicates	474	281	932	551.5	285	436.5	234	173.5	196.5	308	436.5	240.5	243.5	217.5	359	568	478.5	469
		**average all**	**382.4722**																	
A10L	MPXV_ZAI1979_126		547	341	260	286	249	360	475	373	361	664	285	429	530	361	254	432	415	253
	MPXV_ZAI1979_126		644	377	249	261	357	380	418	327	332	699	304	462	359	314	230	444	369	225
	MPXV_ZAI1979_126		390	268	160	250	281	223	335	322	316	534	235	322	314	262	186	353	263	312
	MPXV_ZAI1979_126		541	255	180	267	208	273	327	343	258	568	327	430	328	194	196	406	265	295
	MPXV_ZAI1979_126		149	327	260	177	192	274	273	174	241	599	194	259	280	201	154	229	154	227
	MPXV_ZAI1979_126		171	491	262	174	188	188	261	167	217	573	207	316	220	200	184	238	154	247
		average of replicates	407	343.1667	228.5	235.8333	245.8333	283	348.1667	284.3333	287.5	606.1667	258.6667	369.6667	338.5	255.3333	200.6667	350.3333	270	259.8333
		**average all**	**309.5833**																	
A26L	MPXV_ZAI1979_146		169	263	274	220	266	187	317	314	254	251	178	267	317	329	266	316	503	318
	MPXV_ZAI1979_146		132	237	303	234	393	155	362	321	320	175	193	267	252	317	260	335	440	314
		average of replicates	150.5	250	288.5	227	329.5	171	339.5	317.5	287	213	185.5	267	284.5	323	263	325.5	471.5	316
		**average all**	**278.3056**																	
A27L	MPXV_ZAI1979_147		273	884	350	378	169	658	169	460	163	635	573	303	163	256	349	499	339	214
	MPXV_ZAI1979_147		267	950	324	360	186	754	192	316	182	612	664	353	170	185	298	496	319	194
		average of replicates	270	917	337	369	177.5	706	180.5	388	172.5	623.5	618.5	328	166.5	220.5	323.5	497.5	329	204
		**average all**	**379.3611**																	
A27L	MPXV-WRAIR132		141	365	533	347	164	303	190	497	175	992	313	247	227	291	353	377	225	264
	MPXV-WRAIR132		133	347	483	321	164	273	208	496	177	1012	338	175	192	294	432	388	232	270
		average of replicates	137	356	508	334	164	288	199	496.5	176	1002	325.5	211	209.5	292.5	392.5	382.5	228.5	267
		**average all**	**331.6389**																	
D8L	MPXV_ZAI1979_110		281	164	144	114	160	151	125	94	140	189	129	188	144	139	124	170	111	129
	MPXV_ZAI1979_110		185	164	145	115	152	144	78	87	121	195	136	194	149	139	122	159	120	143
		average of replicates	233	164	144.5	114.5	156	147.5	101.5	90.5	130.5	192	132.5	191	146.5	139	123	164.5	115.5	136
		**average all**	**145.6667**																	
D8L	MPXV-WRAIR099		205	762	634	537	562	829	754	650	754	591	590	824	841	721	530	832	573	645
	MPXV-WRAIR099		161	657	658	581	599	578	770	669	709	585	568	762	977	781	481	831	551	662
		average of replicates	183	709.5	646	559	580.5	703.5	762	659.5	731.5	588	579	793	909	751	505.5	831.5	562	653.5
		**average all**	**650.3889**																	
D11L	MPXV_ZAI1979_113		2675	706	347	499	462	1325	490	455	615	6	1168	556	349	377	462	382	592	503
	MPXV_ZAI1979_113		3090	643	430	430	468	1426	483	446	610	14	1152	614	417	22	537	350	539	432
		average of replicates	2882.5	674.5	388.5	464.5	465	1375.5	486.5	450.5	612.5	10	1160	585	383	199.5	499.5	366	565.5	467.5
		**average all**	**668.6667**																	
F17R	MPXV_ZAI1979_052		5387	9703	4712	7801	5797	10813	2877	1438	2781	3727	10813	5387	1496	3967	7164	1651	2506	2359
	MPXV_ZAI1979_052		5809	10813	4471	7164	5809	9703	3405	1453	2867	3635	9703	5277	1453	3422	7154	1536	2558	2383
		average of replicates	5598	10258	4591.5	7482.5	5803	10258	3141	1445.5	2824	3681	10258	5332	1474.5	3694.5	7159	1593.5	2532	2371
		**average all**	**4972.056**																	
H3L	MPXV_ZAI1979_098		444	359	270	348	339	272	396	410	294	265	305	327	521	406	354	490	430	314
	MPXV_ZAI1979_098		500	353	313	361	312	259	388	507	335	237	266	317	499	477	296	504	390	362
		average of replicates	472	356	291.5	354.5	325.5	265.5	392	458.5	314.5	251	285.5	322	510	441.5	325	497	410	338
		**average all**	**367.2222**																	

H3L	MPXV-WRAIR087			963	614	540	488	704	795	828	649	824	573	700	767	699	662	649	612	733	592
	MPXV-WRAIR087			734	615	509	496	665	657	781	649	754	574	669	798	701	684	649	608	706	614
			average of replicates	848.5	614.5	524.5	492	684.5	726	804.5	649	789	573.5	684.5	782.5	700	673	649	610	719.5	603
			**average all**	**673.75**																	
L4R	MPXV_ZAI1979_088			2555	2794	3852	2569	3967	2437	4248	3492	3927	3762	3055	3502	3967	4248	2781	4248	2608	4016
	MPXV_ZAI1979_088			2468	2531	3715	3327	3715	2781	4143	3405	3715	3492	2940	3492	3927	3927	2877	5062	2863	3762
			average of replicates	2511.5	2662.5	3783.5	2948	3841	2609	4195.5	3448.5	3821	3627	2997.5	3497	3947	4087.5	2829	4655	2735.5	3889
			**average all**	**3449.167**																	
A16L	MPXV_ZAI1979_133			127	77	106	123	94	156	133	139	120	104	125	123	110	97	132	129	141	91
	MPXV_ZAI1979_133			172	96	96	87	57	143	132	134	96	106	128	108	115	112	112	129	131	97
			average of replicates	149.5	86.5	101	105	75.5	149.5	132.5	136.5	108	105	126.5	115.5	112.5	104.5	122	129	136	94
			**average all**	**116.0556**																	
G4L	MPXV_ZAI1979_078			110	124	129	106	145	144	85	159	159	154	108	164	136	115	133	131	128	145
	MPXV_ZAI1979_078			155	99	122	113	114	71	106	142	164	143	69	106	132	123	134	134	136	144
			average of replicates	132.5	111.5	125.5	109.5	129.5	107.5	95.5	150.5	161.5	148.5	88.5	135	134	119	133.5	132.5	132	144.5
			**average all**	**127.2778**																	
E10R	MPXV_ZAI1979_063			140	103	125	106	123	103	129	83	70	84	96	127	77	137	80	111	97	84
	MPXV_ZAI1979_063			118	104	125	94	136	121	121	103	75	92	56	125	84	120	77	113	96	84
			average of replicates	129	103.5	125	100	129.5	112	125	93	72.5	88	76	126	80.5	128.5	78.5	112	96.5	84
			**average all**	**103.3056**																	
E10R	MPXV-WRAIR052			254	286	360	320	268	497	265	325	406	268	378	418	322	341	328	361	372	356
	MPXV-WRAIR052			350	330	439	374	262	446	338	361	341	260	329	327	327	343	267	346	314	321
	MPXV-WRAIR052			3508	504	406	432	350	401	365	429	497	274	549	442	417	354	371	428	546	367
	MPXV-WRAIR052			3159	367	475	349	319	458	354	370	415	262	538	504	370	368	349	401	474	357
			average of replicates	1817.75	371.75	420	368.75	299.75	450.5	330.5	371.25	414.75	266	448.5	422.75	359	351.5	328.75	384	426.5	350.25
			**average all**	**454.5694**																	
A3L	MPXV_ZAI1979_119			134	329	348	414	284	341	262	289	314	649	365	132	472	367	372	264	300	483
	MPXV_ZAI1979_119			161	343	371	445	238	333	187	310	305	580	328	203	460	320	391	267	262	452
			average of replicates	147.5	336	359.5	429.5	261	337	224.5	299.5	309.5	614.5	346.5	167.5	466	343.5	381.5	265.5	281	467.5
			**average all**	**335.4167**																	
A13L	MPXV_ZAI1979_129			1513	139	65	92	117	160	112	139	108	123	110	122	120	62	106	97	82	155
	MPXV_ZAI1979_129			1994	149	83	70	103	164	120	126	115	126	127	124	110	84	118	110	77	169
			average of replicates	1753.5	144	74	81	110	162	116	132.5	111.5	124.5	118.5	123	115	73	112	103.5	79.5	162
			**average all**	**205.3056**																	
A14L	MPXV_ZAI1979_130			47	72	97	72	48	68	65	59	62	97	65	45	80	81	76	62	76	65
	MPXV_ZAI1979_130			58	66	92	77	67	118	52	44	62	105	68	60	69	58	73	59	94	63
			average of replicates	52.5	69	94.5	74.5	57.5	93	58.5	51.5	62	101	66.5	52.5	74.5	69.5	74.5	60.5	85	64
			**average all**	**70.05556**																	
A42R	MPXV_ZAI1979_161			135	143	138	104	114	261	101	117	125	121	162	144	126	91	136	103	142	129
	MPXV_ZAI1979_161			47	136	136	120	160	254	118	127	127	113	262	101	128	121	132	104	132	140
			average of replicates	91	139.5	137	112	137	257.5	109.5	122	126	117	212	122.5	127	106	134	103.5	137	134.5
			**average all**	**134.7222**																	
I2L	MPXV_ZAI1979_068			58	277	107	111	75	155	71	113	120	106	105	77	87	105	130	82	139	94
	MPXV_ZAI1979_068			52	124	106	126	92	127	71	112	144	108	105	83	101	106	123	87	137	90
			average of replicates	55	200.5	106.5	118.5	83.5	141	71	112.5	132	107	105	80	94	105.5	126.5	84.5	138	92
			**average all**	**108.5**																	

A16L	MPXV_ZAI1979_133		314	112.5	103.5	225.5	125	125	135.5	152.5	150	138	130	120	117	102.5	86.5	114.5	158	112.5
	MPXV_ZAI1979_133		287.5	122	90	23.5	132	119	124	129.5	82.5	117	99	117	109	79.5	139.5	139.5	99	107
		average of replicates	300.75	117.25	96.75	124.5	128.5	122	129.75	141	116.25	127.5	114.5	118.5	113	91	113	127	128.5	109.75
		**average all**	**128.86111**																	
G4L	MPXV_ZAI1979_078		299	193.5	162	243.5	87.5	148.5	93.5	115	75.5	257.5	128	134	189	275	140.5	153	313	181.5
	MPXV_ZAI1979_078		288.5	169.5	152	341	117.5	146	110	141.5	128	229	144	142.5	164	226.5	141	154.5	313.5	193.5
		average of replicates	293.75	181.5	157	292.25	102.5	147.25	101.75	128.25	101.75	243.25	136	138.25	176.5	250.75	140.75	153.75	313.25	187.5
		**average all**	**180.33333**																	
E10R	MPXV_ZAI1979_063		40	118	125	81	134	118	105	122	198.5	142	142	107.5	100.5	141.5	85	107.5	528.5	109
	MPXV_ZAI1979_063		190	85.5	111	95.5	132.5	105	109	104	292.5	110.5	110.5	113.5	105	87	102.5	117.5	65	107.5
		average of replicates	115	101.75	118	88.25	133.25	111.5	107	113	245.5	126.25	126.25	110.5	102.75	114.25	93.75	112.5	296.75	108.25
		**average all**	**129.13889**																	
E10R	MPXV-WRAIR052		695.5	333	310	515	333.5	390.5	375.5	378.5	347.5	503.5	298	306	360	440	458.5	435	776	460.5
	MPXV-WRAIR052		598	327	362.5	564.5	340	395	384.5	362.5	307	383.5	303	284	354	402.5	411	428.5	658	469
	MPXV-WRAIR052		837	395	381	647	375.5	468	531	303.5	447.5	448	444	348.5	398	550.5	452.5	490.5	752.5	417
	MPXV-WRAIR052		510.5	379	401	591	407.5	401	512	362	439.5	384.5	443.5	307	405.5	604	440	509	824	409
		average of replicates	660.25	358.5	363.625	579.375	364.125	413.625	450.75	351.625	385.375	429.875	372.125	311.375	379.375	499.25	440.5	465.75	752.625	438.875
		**average all**	**445.38889**																	
A3L	MPXV_ZAI1979_119		242.5	270	428.5	360.5	467	539	590	435.5	266	559.5	399	457.5	380	322.5	419	547.5	354.5	732.5
	MPXV_ZAI1979_119		253.5	232	464	328.5	450.5	466.5	617.5	417	223.5	483	370	466.5	340	310.5	428.5	439.5	203.5	762
		average of replicates	248	251	446.25	344.5	458.75	502.75	603.75	426.25	244.75	521.25	384.5	462	360	316.5	423.75	493.5	279	747.25
		**average all**	**417.43056**																	
A13L	MPXV_ZAI1979_129		270	106	139.5	288	81	87.5	110.5	170	113.5	113.5	125	105	270	177	153.5	95.5	225.5	178
	MPXV_ZAI1979_129		357.5	110	134	169.5	82	95.5	117	176	70.5	109	90	102.5	240	205	144.5	91	128	168.5
		average of replicates	313.75	108	136.75	228.75	81.5	91.5	113.75	173	92	111.25	107.5	103.75	255	191	149	93.25	176.75	173.25
		**average all**	**149.98611**																	
A14L	MPXV_ZAI1979_130		260	68.5	59	105.5	51	102	59.5	70	51.5	63.5	69.5	100	91.5	54	74	78.5	68	102.5
	MPXV_ZAI1979_130		47	69	83	83	59.5	104.5	72.5	81	62.5	112	70.5	91.5	83	80	61	84	145.5	99
		average of replicates	153.5	68.75	71	94.25	55.25	103.25	66	75.5	57	87.75	70	95.75	87.25	67	67.5	81.25	106.75	100.75
		**average all**	**83.805556**																	
A42R	MPXV_ZAI1979_161		307	130	178	242.5	102	221	159	145	164	169	104	166.5	105.5	249	172.5	111	229	136.5
	MPXV_ZAI1979_161		324.5	109	183.5	255.5	79.5	192.5	150.5	150.5	168	160	107.5	163.5	112	294.5	227.5	88.5	337.5	149.5
		average of replicates	315.75	119.5	180.75	249	90.75	206.75	154.75	147.75	166	164.5	105.75	165	108.75	271.75	200	99.75	283.25	143
		**average all**	**176.26389**																	
I2L	MPXV_ZAI1979_068		255.5	103	97	98.5	86.5	103	107.5	123.5	67.5	146.5	112	124	139	127.5	86	155.5	78.5	134
	MPXV_ZAI1979_068		441.5	87	93.5	168.5	81	93	90	127.5	74	135	117	104	124	151	99	100	98.5	141
		average of replicates	348.5	95	95.25	133.5	83.75	98	98.75	125.5	70.75	140.75	114.5	114	131.5	139.25	92.5	127.75	88.5	137.5
		**average all**	**124.18056**																	

Vaccinia				Non-Human Primates Day 28 (IgG)						Non-Human Primates Day -1 (IgM)					Non-Human Primates Day 6 (IgM)					Human Controls (IgG)					Human Post Vaccination (IgG)			
Ortholog	Database ID			3	6	7	11	15		3	6	7	11		3	6	7	11		1	2	3	4		1	2	3	4
A4L	MPXV_ZAI1979_120			2494	9703	2846	5171	829		299.5	344.5	560	302.5		2257	829.5	2624.5	1832.5		6554	923.5	1387.5	718.5		3352	3250	3391	22244
	MPXV_ZAI1979_120			2468	10813	2712	5144	841		307.5	320	581	379.5		2174.5	856	2498	1771		5894.5	951	1199	694.5		2209	2979.5	3073.5	16434
		average of replicates	average of replicates	2481	10258	2779	5157.5	835	average of replicates	303.5	332.25	570.5	341	average of replicates	2215.75	842.75	2561.25	1801.75	average of replicates	6224.25	937.25	1293.25	706.5	average of replicates	2780.5	3114.75	3232.25	19339
		**average all**	**average all**	**4302.1**					**average all**	**386.8125**				**average all**	**1855.375**				**average all**	**2290.313**				**average all**	**7116.625**			
A4L	MPXV-WRAIR109			2431	5797	2620	4712	764		313.5	475	689	442.5		2065.5	733.5	2051	1361		5535	1004.5	1121.5	767.5		4265.5	6535.5	6017	29564.5
	MPXV-WRAIR109			2437	5809	2655	4602	706		280	482.5	665.5	455		2116.5	597.5	2589	1350		5541	983	1199.5	1116.5		4778	5155.5	5947.5	25159
		average of replicates	average of replicates	2434	5803	2637.5	4657	735	average of replicates	296.75	478.75	677.25	448.75	average of replicates	2091	665.5	2320	1355.5	average of replicates	5538	993.75	1160.5	942	average of replicates	4521.75	5845.5	5982.25	27361.75
		**average all**	**average all**	**3253.3**					**average all**	**475.375**				**average all**	**1608**				**average all**	**2158.563**				**average all**	**10927.8125**			
A10L	MPXV_ZAI1979_126			3475	2846	3073	2867	2386		171.5	198	223	210		386	296.5	1012.5	462		8046.5	7374	588.5	1097		11469.5	28811	23615.5	14121
	MPXV_ZAI1979_126			3492	2808	3090	2781	2434		131	182.5	182.5	173		393	290.5	423	425.5		8789.5	7064.5	541.5	1158		11898	28059.5	22536	13719.5
	MPXV_ZAI1979_126			3111	2530	2964	2569	2467		172.5	142	189	222		477	342.5	344	467.5		9201	8289	536	846		10254	26162.5	18226	12059
	MPXV_ZAI1979_126			3159	2506	2808	2751	2558		195.5	172.5	154	199.5		483	316	273	421		13059.5	7549	546	869		11153.5	26681.5	16434	10689
	MPXV_ZAI1979_126			2794	2431	2738	2383	2129		118	58	78.5	94		257.5	154.5	133	174		6248.5	3701	308	534		12059	22536	13473	13399.5
	MPXV_ZAI1979_126			2751	2437	2751	2355	2089		96	66	82	93.5		218	139	176	419		6075	3503	328	485		12227.5	23615.5	14676.5	12865.5
		average of replicates	average of replicates	3130.333	2593	2904	2617.667	2343.833	average of replicates	147.41667	136.5	151.5	165.33333	average of replicates	369.08333	256.5	393.58333	394.83333	average of replicates	8570	6246.75	474.66667	831.5	average of replicates	11510.25	25977.66667	18160.16667	12808.91667
		**average all**	**average all**	**2717.767**					**average all**	**150.1875**				**average all**	**353.5**				**average all**	**4030.729**				**average all**	**17114.25**			
A26L	MPXV_ZAI1979_146			3135	2964	2437	2277	5387		120	156	138.5	139.5		457.5	293.5	440	452		6975	5312.5	965.5	583		14121	22378	9639	7133.5
	MPXV_ZAI1979_146			3104	2958	2383	2324	5144		124.5	171.5	149.5	134.5		455	294	436	401.5		6878.5	5465.5	1164	526		14676.5	22244	10254	6842
		average of replicates	average of replicates	3119.5	2961	2410	2300.5	5265.5	average of replicates	122.25	163.75	144	137	average of replicates	456.25	293.75	438	426.75	average of replicates	6926.75	5389	1064.75	554.5	average of replicates	14398.75	22311	9946.5	6987.75
		**average all**	**average all**	**3211.3**					**average all**	**141.75**				**average all**	**403.6875**				**average all**	**3483.75**				**average all**	**13411**			
A27L	MPXV_ZAI1979_147			5062	3715	4003	1386	898		910	876.5	1261.5	954		2498	3287	2650	2674.5		8289	6277.5	8098.5	1569		13819.5	3801	3113.5	2035
	MPXV_ZAI1979_147			4602	3111	3967	1325	938		883.5	1248	1161.5	1047.5		2610.5	3230.5	2783.5	2589		8575.5	6457.5	8789.5	1366.5		13122	3092.5	3288.5	1720.5
		average of replicates	average of replicates	4832	3413	3985	1355.5	918	average of replicates	896.75	1062.25	1211.5	1000.75	average of replicates	2554.25	3258.75	2716.75	2631.75	average of replicates	8432.25	6367.5	8444	1467.75	average of replicates	13470.75	3446.75	3201	1877.75
		**average all**	**average all**	**2900.7**					**average all**	**1042.8125**				**average all**	**2790.375**				**average all**	**6177.875**				**average all**	**5499.0625**			
A27L	MPXV-WRAIR132			5387	3422	4143	1246	701		495	1000.5	824.5	746.5		1732.5	1980.5	2295.5	2150.5		16237.5	6945.5	8575.5	1609		25159	4778	10689	2788.5
	MPXV-WRAIR132			5277	3405	4268	1263	619		466	850	832.5	757		1889	2389	2022	2053.5		16541.5	6734.5	8289	1673.5		18226	3980	10316.5	2797.5
		average of replicates	average of replicates	5332	3413.5	4205.5	1254.5	660	average of replicates	480.5	925.25	828.5	751.75	average of replicates	1810.75	2184.75	2158.75	2102	average of replicates	16389.5	6840	8432.25	1641.25	average of replicates	21692.5	4379	10502.75	2793
		**average all**	**average all**	**2973.1**					**average all**	**746.5**				**average all**	**2064.0625**				**average all**	**8325.75**				**average all**	**9841.8125**			
D8L	MPXV_ZAI1979_110			2337	2863	2376	2583	2738		146	155	153	126.5		495.5	975	431.5	3262		4940.5	532.5	587.5	816		1431.5	2833.5	2105.5	3313.5
	MPXV_ZAI1979_110			2314	3073	2359	2555	2586		140.5	140.5	134.5	161		550.5	993.5	405	3346.5		5212.5	472	522	826.5		1442	2765.5	2052	2375.5
		average of replicates	average of replicates	2325.5	2968	2367.5	2569	2662	average of replicates	143.25	147.75	143.75	143.75	average of replicates	523	984.25	418.25	3304.25	average of replicates	5076.5	502.25	554.75	821.25	average of replicates	1436.75	2799.5	2078.75	2844.5
		**average all**	**average all**	**2578.4**					**average all**	**144.625**				**average all**	**1307.4375**				**average all**	**1738.688**				**average all**	**2289.875**			
D8L	MPXV-WRAIR099			2706	4268	3104	3327	3927		489.5	437.5	463	490		1396	1679.5	897	5340		5917.5	1761	1534	1455		2537	3330	3847.5	3925.5
	MPXV-WRAIR099			2863	4248	3111	3159	3762		578	657.5	469.5	372.5		1390.5	1976.5	1230.5	4873		5979.5	1809	1429	1242		2436.5	3018	3920.5	3581.5
		average of replicates	average of replicates	2784.5	4258	3107.5	3243	3844.5	average of replicates	533.75	547.5	466.25	431.25	average of replicates	1393.25	1828	1063.75	5106.5	average of replicates	5948.5	1785	1481.5	1348.5	average of replicates	2486.75	3174	3884	3753.5
		**average all**	**average all**	**3447.5**					**average all**	**494.6875**				**average all**	**2347.875**				**average all**	**2640.875**				**average all**	**3324.5625**			
D11L	MPXV_ZAI1979_113			252	0	319	799	895		961	1156	1146	1002.5		909.5	592.5	1990	1396		1223	1581	1921.5	610.5		1079	1552.5	1346.5	1780.5
	MPXV_ZAI1979_113			256	6	327	764	1074		981.5	1365	1094.5	1300.5		1077.5	598	1936	1358.5		1193	1569	1676.5	577.5		1128	1476	1332.5	1464
		average of replicates	average of replicates	254	3	323	781.5	984.5	average of replicates	971.25	1260.5	1120.25	1151.5	average of replicates	993.5	595.25	1963	1377.25	average of replicates	1208	1575	1799	594	average of replicates	1103.5	1514.25	1339.5	1622.25
		**average all**	**average all**	**469.2**					**average all**	**1125.875**				**average all**	**1232.25**				**average all**	**1294**				**average all**	**1394.875**			
F17R	MPXV_ZAI1979_052			3762	2794	2468	2437	10813		5466	7975	9153	7975		8761	12819	12819	12819		7374	10257	29556	14852		2152	3608	4639.5	3463.5
	MPXV_ZAI1979_052			3635	2860	2434	2337	9703		9153	5950	7975	5660.5		7770	10546.5	10546.5	10546.5		6945.5	10531	31592	7582		3250	3201.5	5155.5	3639.5
		average of replicates	average of replicates	3698.5	2827	2451	2387	10258	average of replicates	7309.5	6962.5	8564	6817.75	average of replicates	8265.5	11682.75	11682.75	11682.75	average of replicates	7159.75	10394	30574	11217	average of replicates	2701	3404.75	4897.5	3551.5
		**average all**	**average all**	**4324.3**					**average all**	**7413.4375**				**average all**	**10828.438**				**average all**	**14836.19**				**average all**	**3638.6875**			
H3L	MPXV_ZAI1979_098			2781	2531	2604	2675	2437		333	194	335.5	274.5		1447	747.5	1507	1184.5		7981	3781	950.5	827.5		22536	10204.5	35615	5947.5
	MPXV_ZAI1979_098			2569	2494	2583	2604	2337		315.5	189.5	338	302.5		1420	828.5	1350	1398		8098.5	4029.5	744	811.5		26162.5	8489	31234.5	4148
		average of replicates	average of replicates	2675	2512.5	2593.5	2639.5	2387	average of replicates	324.25	191.75	336.75	288.5	average of replicates	1433.5	788	1428.5	1291.25	average of replicates	8039.75	3905.25	847.25	819.5	average of replicates	24349.25	9346.75	33424.75	5047.75
		**average all**	**average all**	**2561.5**					**average all**	**285.3125**				**average all**	**1235.3125**				**average all**	**3402.938**				**average all**	**18042.125**			
H3L	MPXV-WRAIR087			3502	5144	5171	5387	2712		581	691	528.5	503		2064	2584	1608.5	1842.5		10531	5353.5	2002	1163		33786.5	6724	48387	3318
	MPXV-WRAIR087			3508	5170	5170	5663	2751		592	511	592	534.5		1980.5	2488	1537.5	1947.5		12084	5423.5	2002.5	1170		28059.5	6140.5	40330.5	2765.5
		average of replicates	average of replicates	3505	5157	5170.5	5525	2731.5	average of replicates	586.5	601	560.25	518.75	average of replicates	2022.25	2536	1573	1895	average of replicates	11307.5	5388.5	2002.25	1166.5	average of replicates	30923	6432.25	44358.75	3041.75
		**average all**	**average all**	**4417.8**					**average all**	**566.625**				**average all**	**2006.5625**				**average all**	**4966.188**				**average all**	**21188.9375**			
L4R	MPXV_ZAI1979_088			2359	2738	5277	2131	2085		1486.5	1499	1704.5	1704.5		1754	1329.5	2163.5	1256		2184.5	4460	2708	1640.5		3318	12865.5	1941	2956.5
	MPXV_ZAI1979_088			2376	2655	4761	2121	1994		1217.5	1300.5	1781.5	1340.5		1402.5	843	2127.5	1344.5		2376.5	4518.5	2820.5	1654.5		3368	10535	2046.5	2678
		average of replicates	average of replicates	2367.5	2696.5	5019	2126	2039.5	average of replicates	1352	1399.75	1743	1522.5	average of replicates	1578.25	1086.25	2145.5	1300.25	average of replicates	2280.5	4489.25	2764.25	1647.5	average of replicates	3343	11700.25	1993.75	2817.25
		**average all**	**average all**	**2849.7**					**average all**	**1504.3125**				**average all**	**1527.5625**				**average all**	**2795.375**				**average all**	**4963.5625**			
A16L	MPXV_ZAI1979_133			92	94	119	109	122		106	172.5	108.5	114.5		252.5	226.5	258.5	255		457	298	499.5	366.5		469.5	225	488.5	263
	MPXV_ZAI1979_133			84	106	127	123	116		112.5	173	113.5	97.5		255	241.5	252.5	198.5		394	286.5	459	386.5		556	226	548.5	270
		average of replicates	average of replicates	88	100	123	116	119	average of replicates	109.25	172.75	111	106	average of replicates	253.75	234	255.5	226.75	average of replicates	425.5	292.25	479.25	376.5	average of replicates	512.75	225.5	518.5	266.5
		**average all**	**average all**	**109.2**					**average all**	**124.75**				**average all**	**242.5**				**average all**	**393.375**				**average all**	**380.8125**			
G4L	MPXV_ZAI1979_078			813	540	1260	1453	453		204.5	135	185	240.5		610	1811.5	488	485.5		230	682.5	182	317.5		48.5	529	829.5	81
	MPXV_ZAI1979_078			795	635	1263	1356	477		189	139.5	204	242.5		626.5	1287	477.5	395.5		248	578.5	183	335.5		55	464.5	766	96.5
		average of replicates	average of replicates	804	587.5	1261.5	1404.5	465	average of replicates	196.75	137.25	194.5	241.5	average of replicates	618.25	1549.25	482.75	440.5	average of replicates	239	630.5	182.5	326.5	average of replicates	51.75	496.75	797.75	88.75
		**average all**	**average all**	**904.5**					**average all**	**192.5**				**average all**	**772.6875**				**average all**	**344.625**				**average all**	**358.75**			
E10R	MPXV_ZAI1979_063			114	81	91	94	72		93.5	120	117.5	96.5		251.5	405.5	331	234.5		465.5	337	275.5	903		621	311	537	312
	MPXV_ZAI1979_063			112	81	114	77	84		83.5	100.5	120.5	110.5		242	419.5	237.5	287		476	313	304	1022.5		540	340.5	519	269.5
		average of replicates	average of replicates	113	81	102.5	85.5	78	average of replicates	88.5	110.25	119	103.5	average of replicates	246.75	412.5	284.25	260.75	average of replicates	470.75	325	289.75	962.75	average of replicates	580.5	325.75	528	290.75
		**average all**	**average all**	**92**					**average all**	**105.3125**				**average all**	**301.0625**				**average all**	**512.0625**				**average all**	**431.25**			
E10R	MPXV-WRAIR052			347	281	202	262	373		746.5	676	878	727.5		1226.5	1996.5	1639	1261.5		1436	1184.5	1744.5	3503		1786	583	912	659
	MPXV-WRAIR052			333	270	186	269	356		567.5	869	879	747		1184.5	2004	1679.5	1534.5		1188.5	1113	1455	3056.5		1541	620	869.5	601.5
	MPXV-WRAIR052			328	297	310	324	468		726	895	626.5	715.5		1431.5	1986.5	1830.5	1600		1700	1402.5	1272.5	3920.5		1552.5	621.5	667	734.5
	MPXV-WRAIR052			338	303	268	327	480		700.5	792.5	806	765		1483.5	1940.5	1534	1556		1722.5	1489	1318.5	4029.5		1672.5	656	733	597.5
		average of replicates	average of replicates	336.5	287.75	241.5	295.5	419.25	average of replicates	685.125	808.125	797.375	738.75	average of replicates	1457.5	1963.5	1682.25	1578	average of replicates	1711.25	1445.75	1295.5	3975	average of replicates	1638	620.125	795.375	648.125
		**average all**	**average all**	**316.1**					**average all**	**757.34375**				**average all**	**1670.3125**				**average all**	**2106.875**				**average all**	**925.40625**			
A3L	MPXV_ZAI1979_119			206	376	341	1168	310		273	113	225.5	232		631.5	466.5	535.5	668.5		1656.5	728.5	1198.5	786.5		1883	2527	702	1028
	MPXV_ZAI1979_119			206	327	348	1182	235		273	182.5	259.5	229		577.5	457.5	574	524		1686	705	1242	710.5		1977	2253	724	991.5
		average of replicates	average of replicates	206	351.5	344.5	1175	272.5	average of replicates	434.44792	147.75	242.5	230.5	average of replicates	604.5	462	554.75	596.25	average of replicates	1671.25	716.75	1220.25	748.5	average of replicates	1930	2390	713	1009.75
		**average all**	**average all**	**469.9**					**average all**	**263.79948**				**average all**	**554.375**				**average all**	**1089.188**				**average all**	**1510.6875**			
A13L	MPXV_ZAI1979_129			432	1899	549	2128	643		192.5	188.5	232	197		462.5	412	487	442		471.5	304	330.5	248		86.5	224	1202.5	630
	MPXV_ZAI1979_129			391	1994	616	2320	551		171.5	171.5	226.5	199.5		428.5	343.5	483	475		462.5	306	321	174		101.5	199	1231	766
		average of replicates	average of replicates	411.5	1946.5	582.5	2224	597	average of replicates	209.26649	180	229.25	198.25	average of replicates	445.5	377.75	485	458.5	average of replicates	467	305	325.75	211	average of replicates	94	211.5	1216.75	698
		**average all**	**average all**	**1152.3**					**average all**	**204.19162**				**average all**	**441.6875**				**average all**	**327.1875**				**average all**	**555.0625**			
A14L	MPXV_ZAI1979_130			99	214	52	72	57		74.5	32.5	61	75.5		189.5	178	184.5	176		426.5	369.5	237.5	237.5		582	659	991.5	476.5
	MPXV_ZAI1979_130			107	206	56	66	61		74.5	27	51.5	68		179.5	161.5	179.5	103.5		386	303	284.5	268.5		587.5	664.5	1056.5	492.5
		average of replicates	average of replicates	103	210	54	69	59	average of replicates	117.73054	29.75	56.25	71.75	average of replicates	184.5	169.75	182	139.75	average of replicates	406.25	336.25	261	253	average of replicates	584.75	661.75	1024	484.5

		**average all**	**average all**	**99**					**average all**	**68.870135**					**average all**	**169**					**average all**	**314.125**					**average all**	**688.75**			
A42R	MPXV_ZAI1979_161			115	96	132	132	164		123	133	134	113		275.5	393	344.5	299.5		373.5	612.5	413.5	573.5		415	194.5	627.5	323
	MPXV_ZAI1979_161			79	100	123	110	249		101	151	123	125		220	396.5	308	339.5		369	578	369.5	590.5		412	189	533	290.5
		average of replicates	average of replicates	97	98	127.5	121	206.5	average of replicates	97.623378	142	128.5	119	average of replicates	247.75	394.75	326.25	319.5	average of replicates	371.25	595.25	391.5	582	average of replicates	413.5	191.75	580.25	306.75
		**average all**	**average all**	**130**					**average all**	**121.78084**					**average all**	**322.0625**					**average all**	**485**					**average all**	**373.0625**			
I2L	MPXV_ZAI1979_068			162	124	83	103	73		142	110.5	140.5	149.5		399.5	726.5	425	393		348	204.5	271	508		141	110	161	160
	MPXV_ZAI1979_068			143	122	72	104	115		152.5	85.5	152.5	147		492.5	732	386	405		361	211.5	275	493.5		146.5	111.5	160	183
		average of replicates	average of replicates	152.5	123	77.5	103.5	94	average of replicates	138.76028	98	146.5	148.25	average of replicates	446	729.25	405.5	399	average of replicates	354.5	208	273	500.75	average of replicates	143.75	110.75	160.5	171.5
		**average all**	**average all**	**110.1**					**average all**	**132.87757**					**average all**	**494.9375**					**average all**	**334.0625**					**average all**	**146.625**			
